# Hand-assisted laparoscopic nephrectomy in a high risk overweight donor with left-sided IVC, and previous abdominal surgery

**DOI:** 10.1016/j.ijscr.2019.09.039

**Published:** 2019-09-28

**Authors:** Giuseppe Serena, Javier González, Leonardo E. Garcia, Giselle Guerra, Mahmoud Morsi, Gaetano Ciancio

**Affiliations:** a Department of Surgery, Nassau University Medical Center, East Meadow, NY, USA; b Department of Surgery, University of Miami Miller School of Medicine, Jackson Memorial Hospital, Miami, FL, USA; c Department of Urology, University of Miami Miller School of Medicine, Jackson Memorial Hospital, Miami, FL, USA; d Miami Transplant Institute, University of Miami Miller School of Medicine, Jackson Memorial Hospital, Miami, FL, USA; e Department of Urology, Hospital General Universitario Gregorio Marañón, Madrid, Spain; f Department of Medicine, Division of Nephrology, Miami Transplant Institute, University of Miami Miller School of Medicine, Jackson Memorial Hospital, Miami, FL, USA

**Keywords:** Left-sided inferior vena cava, Venous anomalies, Living donor nephrectomy, Operative complications, Risk factors

## Abstract

**Introduction:**

The extension of donor eligibility criteria represents one of the possible ways to increase the organ shortage, thus decreasing the waiting time for kidney transplantation. Expectedly, this strategy is associated with a growing number of more technically demanding living donor nephrectomy procedures requiring careful assessment, and sound surgical experience in order to avoid intraoperative complications.

**Case presentation:**

After a thorough evaluation through preoperative imaging, we performed a hand-assisted left laparoscopic living donor nephrectomy in a 56 year-old overweight patient with history of prior abdominal surgery, harboring a left-sided inferior vena cava (IVC).

**Discussion/conclusion:**

This case describes our comprehensive approach in this complex surgical scenario to preserve donor safety and provide an optimal kidney graft.

## Highlights

• Surgical planning based on preoperative CTA is necessary in patients with vascular anomalies in order to avoid complications.
• Intrabdominal adhesions are common in patients with previous abdominal surgery and can affect the surgical approach for donor nephrectomy.
• Considering the extension of the donor eligibility criteria, it is expected to see cases with congenital vascular anomalies.

## 1 Introduction

The median time awaiting for adult kidney transplantation continues to rise (estimated increase of 4.3% in patients >5 years in the waiting list for the period 2005–2015) [1]. The extension of donor eligibility criteria represents one of the possible ways to increase the organ shortage, thus decreasing the waiting time for kidney transplantation. Expectedly, this strategy runs parallel to the donor complexity and technical demand of nephrectomy in the living donation setting. In fact, although obesity, vascular anomalies, or prior abdominal surgery were considered ineligible factors for kidney living donation in the past, recent advances has allowed the use of the majority of those kidneys once considered untransplantable [2,3].

Most authors agree that careful assessment focused on each particular case, and certain experience with similar cases are crucial points to avoid misadventures. Actually, the final decision on the donor selection, surgical approach, and kidney graft laterality often relies on the team involved and the accumulated experience of their members rather than on a well established protocol [4].

Herein, we describe our comprehensive approach to an overweight living kidney donor harboring a left-sided IVC, and multiple adhesions secondary to previous abdominal surgery, aiming to provide a rationale for adequate decision-making and counseling, thus empowering surgical safety and optimal kidney graft retrieval.

This study has been reported in line with the SCARE criteria [5].

## 2 Case presentation

**Fig. 1:**
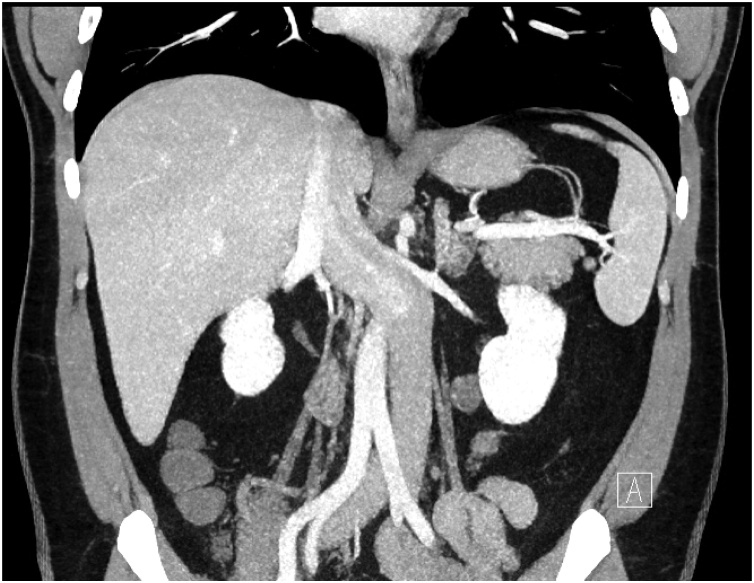
Abdominal CT showing IVC located at the left side of the Aorta.

The patient was a 56-year-old woman who decided to donate a kidney to her husband. Patient was overweight (BMI = 28.4 kg/m^2^) and showed a previous history of cholecystitis and morbid obesity treated with open cholecystectomy and gastric bypass, respectively. She was cleared after full medical assessment, and a careful review of preoperative Computed Tomography angiography (CTA) revealed an infrarenal IVC located at the left side of the Aorta, crossing the midline to its right side at the level of the ostium of a single left renal vein in which both adrenal and gonadal veins drained (Fig. 1). In addition, the length of left single renal artery and vein were measured as 3.88 cm and 3.49 cm (vs 2.68 cm in the right side), respectively. Therefore, left kidney removal deemed best by the living donor selection committee, and hand-assisted laparoscopic approach was selected for the planned nephrectomy.

### 2.1 Description of the procedure

**Fig. 2:**
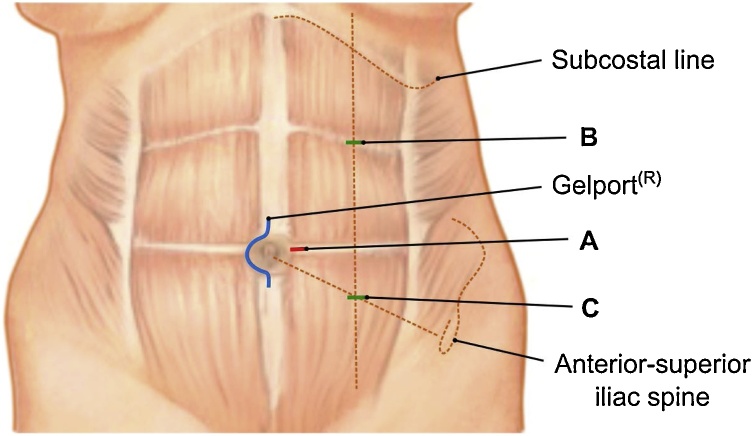
Trocar placement. The hand-assistance device (Gelport®) was placed through a small midline periumbilical surgical wound of 5 cm close to a 10 mm trocar for the optic placement (A). Two additional 5 mm trocars (one placed in the midclavicular line subcostally (B); the other halfway between the umbilicus and the anterior-superior iliac spine (C)), completed the access to the peritoneal cavity.

Written informed consent was obtained prior to surgery from both donor and recipient. After Gelport® and trocar placement (Fig. 2), the abdomen was insufflated to maximally 12 mmHg, showing multiple adhesions extending throughout the abdominal cavity including the left colon, liver, and spleen. The left colon was widely mobilized after gaining enough exposure through extensive laparoscopic adhesiolysis, thus providing a clear surgical field at the major retroperitoneal vessels.

**Fig. 3:**
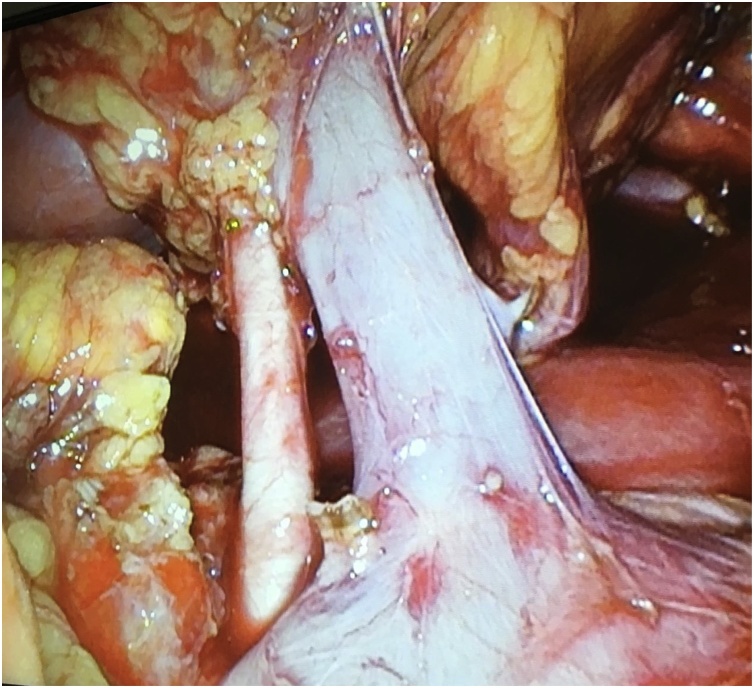
Laparoscopic view of main renal artery and vein isolated before the endovascular stapling. Adrenal and gonadal veins have been dissected and divided using LigaSure® after clip placement. The main renal artery take-off was located posterior to the left-sided IVC.

**Fig. 4:**
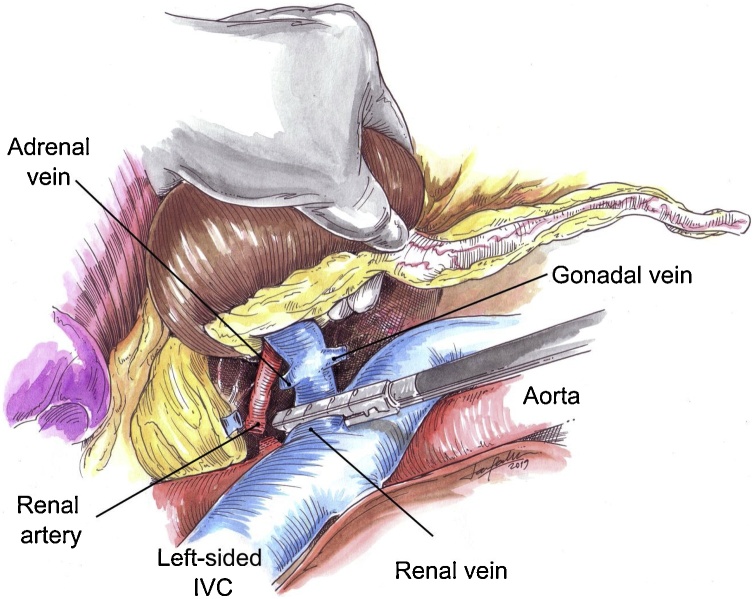
Endoscopic view representation of the left-sided IVC left LDN. The main renal artery and vein were controlled by means of endovascular stapling. Adrenal and gonadal veins draining in the left renal vein were previously divided with LigaSure®.

The infrarenal IVC was identified on the left side of the Aorta and followed upwards until the renal hilum was fully exposed. Gonadal and adrenal veins were dissected and divided using LigaSure®. The ureter was released from its attachments and divided between clips. The main renal vein was located anterior to the course of the main renal artery, and its take-off was placed posterior to the infrarenal IVC (Fig. 3). Both vascular structures were isolated and controlled using an endovascular stapler (Fig. 4).

Blood loss was minimal and the left kidney graft was finally removed through the hand port. Warm ischemic time was 96 s. Status post-procurement was checked upon visualization in the back table, and the kidney graft flushed and preserved with cold Custodiol®-HTK solution. The transplantation procedure was completed in the left recipient´s iliac fossa in a standard fashion showing adequate perfusion and urine output.

Postoperative course of both donor and recipient was uneventful and they were discharged at postoperative day 4 and 5, respectively. Donor and recipient have maintained adequate renal function with no delayed complications and stable serum creatinine (1.13 and 1.31 respectively) at 4 months follow-up.

## 3 Discussion

The chance of getting off the kidney transplant waiting list for the almost 95,000 patients with end stage renal disease awaiting a deceased donor organ in United States was estimated in 1:8 for the year 2018 [6]. Living donation is among the many efforts made to increase organ availability and timely transplantation [7]. However, given the organ scarcity, prospective donor rejection simply on account of technical difficulty does not seem justified nowadays.

Since Ratner et al. [8] description of the first laparoscopic living donor nephrectomy (LDN), the use of minimally invasive surgical approaches offering improved morbidity has become the standard in most transplant centers for procuring living donor kidneys. However, laparoscopic living donor nephrectomy is a technically demanding procedure not exempt of complications given the major vascular dissection, temporary anticoagulation, and rapid kidney extraction requirements to minimize warm ischemia and protect the graft.

Hand-assisted LDN was introduced aiming to combine the advantages of the laparoscopic technique with the quicker and safer kidney retrieval of the open technique (provided by the tactile feedback) [9]. Despite the recommendations of the Kidney Disease Improving Global Outcomes (KDIGO) report [10], in which extensive previous surgery and/or adhesions would justify an open procedure, the rate of conversion to open surgery for hand-assisted LDN in obese donors and patients who have undergone previous abdominal surgery has been reported even slightly lower than that of totally laparoscopic LDN (2.63% vs 4.1%; P = .35) [9,11]. In fact, Kok et al. [12] showed significantly more conversions to open procedure in the presence of intra-abdominal adhesions (0% vs. 10%, P = .005). However, they conclude that these adhesions have no negative effect on the chance of success of the procedure and therefore advise a laparoscopic approach regardless of abdominal surgical history.

Renal vascular anomalies are rare, but if unexpected may represent an important concern in candidates for living kidney donation given its potential for donor and organ damage (vascular injury) in unexperienced hands. Hence, preoperative assessment of pertinent vascular anatomy by means of high resolution cross-sectional imaging is crucial to avoid potential complications, resulting advisable in every potential candidate to better select the most appropriate operation strategy [13].

Left-sided inferior vena cava (LIVC) is caused by the persistence of the left supracardinal and regression of the right supracardinal veins occurred during the complex process of embryogenesis (6–10th weeks of gestation), and represents the second most common anatomical anomaly of the IVC (0.04%–0.5% of population). In the adult form, the left infrarenal portion of the IVC crosses anterior to the aorta at the level of the ostium of the left renal vein, joins the right renal vein, and remains right-sided in its suprarenal portion. However, different degrees of regression and associated ureteric anomalies creates a wide variety of different configurations [14]. Recent reports have confirmed the feasibility and safety of elective LDN in LIVC [11,15,16]. Although Simforoosh et al. [15] reported a relative shortening of the renal veins in LIVC, this fact is not always present. Sometimes the suprarenal and renal portions of the IVC keep their normal anatomic location in the right-side, thus maintaining their length and making not mandatory to choose the right over left kidney for donation. For instance, Kennealey et al. [17] recommended to determine the length of the renal veins preoperatively to decide if the division of the renal vessels via endovascular stapler or Hem-o-lock® was possible in order to avoid troubles during the implantation procedure.

Nevertheless, the debate on the safety, efficacy, and potential advantages of choosing the right or left kidney for LDN is still open [[18], [19], [20]]. In this sense, the first and largest study to compare living donor and recipient outcomes specifically based on laterality in today’s laparoscopic era, showed certain statistical differences in terms of recipient´s rejection rate, delayed graft function, and one-year graft survival in favor of the left side nephrectomy, although the magnitude of difference was extremely small. Regarding donor vascular complications, no significant difference was observed between sides. Therefore, no specific guidelines based on this analysis were finally recommended, leaving the eventual decision to perform a right-sided donor nephrectomy ultimately rely on the unique donor/recipient characteristics along with the surgical team experience/preference [21].

## 4 Conclusion

The extension of the donor eligibility criteria will provide an increasing number of complex vascular cases with previous abdominal surgery. To our knowledge, this is the first case presenting a minimally invasive approach to an overweight living donor with LIVC and simultaneous intra-abdominal adhesions. Careful preoperative evaluation, extended adhesiolysis, wide vascular exposure for adequate visualization, and meticulous hilar dissection were key aspects in avoiding intraoperative complications for the donor and obtaining an optimal kidney graft. Furthermore, both donor and recipient had a successful outcome in terms of renal function.

## Sources of funding

This work was supported by a grant of the “Enrico ed Enrica Sovena” Foundation, Rome, Italy.

## Ethical approval

The case report is exempted from ethical committee.

## Consent

Written informed consent was obtained prior to surgery from both donor and recipient.

## Author contribution

Giuseppe Serena (Drafting Article, Data Collection).

Javier González (Drafting Article, Critical Revision of Article).

Leonardo E Garcia (Drafting Article).

Giselle Guerra (Critical Revision of Article).

Mahmoud Morsi (Critical Revision of Article).

Gaetano Ciancio (Critical Revision of Article, Approval of Article).

## Registration of research studies

Since this is a case report, not a research study, registration is not indicated.

## Guarantor

Gaetano Ciancio MD, MBA, FACS.

## Provenance and peer review

Not commissioned, externally peer-reviewed

## Declaration of Competing Interest

The authors of this manuscript have no conflict of interest to disclose.

## References

[1] Wolfe R.A., Ashby V.B., Milford E.L., Ojo A.O., Ettenger R.E., Agodoa L.Y., Held P.J., Port F.K. (1999). Comparison of mortality in all patients on dialysis, patients on dialysis awaiting transplantation, and recipients of a first cadaveric transplant. N. Engl. J. Med..

[2] Sawinski D., Locke J.E. (2018). Evaluation of kidney donors: core curriculum 2018. Am. J. Kidney Dis..

[3] Ahmadi A.R., Lafranca J.A., Claessens L.A., Imamdi R.M.S., IJzermans J.N.M., Betjes M.G.H., Dor F.J.M.F. (2015). Shifting paradigms in eligibility criteria for live kidney donation: a systematic review. Kidney Int..

[4] Reese P., Feldman H., McBride M., Anderson K., Asch D., Bloom R. (2008). Substantial variation in the acceptance of medically complex live kidney donors across US renal transplant centers. Am. J. Transplant..

[5] Agha R.A., Fowler A.J., Saetta A., Barai I., Rajmohan S., Orgill D.P., for the SCARE Group (2018). The SCARE statement: consensus-based surgical case report guidelines. Int. J. Surg..

[6] United Network for Organ Sharing, https://www.unos.orgdatatransplantTrendswaiting-List-Candidates-by-Organ-Type.

[7] Khan A.S., Shenoy S. (2016). What did we really learn from the collaborative? Is it in our best interest to use “Every organ every time” in kidney transplantation?. Curr. Transpl. Rep..

[8] Ratner L.E., Ciseck L.J., Moore R.G. (1995). Laparoscopic live donor nephrectomy. Transplantation.

[9] Alberts V., Idu M.M., Minnee R.C. (2014). Risk factors for perioperative complications in hand-assisted laparoscopic donor nephrectomy. Prog. Transplant..

[10] Lentine K.L., Kasiske B.L., Levey A.S., Adams P.L., Alberú J., Bakr M.A., Gallon L., Garvey C.A., Guleria S., Li P.K.-T., Segev D.L., Taler S.J., Tanabe K., Wright L., Zeier M.G., Cheung M., Garg A.X. (2017). Summary of kidney disease: improving global outcomes (KDIGO) clinical practice guideline on the evaluation and care of living kidney donors. Transplantation.

[11] Rajabnejad Y., Aliakbarian M., Rajabnejad A., Motie M.R. (2016). Left-sided inferior vena cava encountered during organ retrieval surgery: report of two cases. Int. J. Organ Transplant. Med..

[12] Kok N.F.M., van der Wal J.B.C., Alwayn I.P.J., Tran K.T.C., IJzermans J.N.M. (2008). Laparoscopic kidney donation: the impact of adhesions. Surg. Endosc..

[13] Pandya V.K., Patel A.S., Sutariya H.C., Gandhi S.P. (2016). Evaluation of renal vascular anatomy in live renal donors: role of multi detector computed tomography. Urol. Ann..

[14] González J., Gaynor J.J., Albéniz L.F., Ciancio G. (2017). Inferior vena cava system anomalies: surgical implications. Curr. Urol. Rep..

[15] Simforoosh N., Beigi F.M., Aminsharifi A., Shayaninasab H., Mehrabi S. (2007). Left-sided inferior vena cava found incidentally during laparoscopic donor nephrectomy: report of three cases. J. Endourol..

[16] Radolinski B., Diner E.K., Ghasemian S.R. (2005). Left inferior vena cava in a living kidney donor. J. Urol..

[17] Kennealey P.T., Saidi R.F., Markmann J.F., Ko D.S.C., Kawai T., Yeh H. (2009). Duplicated inferior vena cava—something to consider in the evaluation of a living-donor renal transplant. Dial. Transplant..

[18] Halgrimson W.R., Campsen J., Mandell M.S., Kelly M.A., Kam I., Zimmerman M.A. (2010). Donor complications following laparoscopic compared to hand-assisted living donor nephrectomy: an analysis of the literature. J. Transplant..

[19] Kashiwadate T., Tokodai K., Amada N. (2015). Right versus left retroperitoneoscopic living-donor nephrectomy. Int. Urol. Nephrol..

[20] Liu N.W.R., Wang J., Wang K.J. (2014). Maximizing the donor pool: left versus right laparoscopic live donor nephrectomy-systematic review and meta-analysis. Int. Urol. Nephrol..

[21] Khalil A., Mujtaba M.A., Taber T.E. (2016). Trends and outcomes in right vs. left living donor nephrectomy: an analysis of the OPTN/UNOS database of donor and recipient outcomes—should we be doing more right-sided nephrectomies?. Clin. Transplant..

